# Is self-perception of cardiac symptoms related to the psychological profile of patients? A cross-sectional study of individuals undergoing 24-hour Holter monitoring

**DOI:** 10.31744/einstein_journal/2025AO0742

**Published:** 2025-03-14

**Authors:** Renata Lima Giolo, Guilherme Fenelon, Marcelo Franken, Marcelo Katz

**Affiliations:** 1 Hospital Israelita Albert Einstein São Paulo SP Brazil Hospital Israelita Albert Einstein, São Paulo, SP, Brazil.

**Keywords:** Mental health, Anxiety, Electrocardiography, Arrhythmias, cardiac, Heart diseases, Holtermonitoring, Electrocardiography, ambulatory, Monitoring, physiologic

## Abstract

**Objective:**

This study aimed to examine the presence of psychological characteristics and their association with self-reported cardiac symptoms in individuals undergoing 24-hour Holter monitoring.

**Methods:**

This observational cross-sectional study included 304 individuals who consecutively underwent 24-hour Holter monitoring. Clinical, demographic, and electrocardiographic data were collected. Psycho-behavioral characteristics were assessed using the Hospital Anxiety and Depression Scale and the Type D Scale. Logistic regression models were employed to examine associations between cardiac symptoms and anxiety, depression, distress, and Type D personality traits. Statistical significance was set at p<0.05.

**Results:**

Anxiety, depression, distress, and type D personality traits were observed in 42.7%, 15.1%, 26.3%, and 19% of the participants, respectively. Logistic regression analysis revealed a significant association between the perception of cardiac symptoms and both mild anxiety (odds ratio (OR) = 2.305, 95%CI= 1.098-4.841, p=0.027) and severe anxiety (OR = 9.245, 95%CI= 1.582-54.013, p=0.014) scores. No significant association was found between depression, distress, or Type D personality traits and an increased perception of cardiac symptoms.

**Conclusion:**

A high prevalence of anxiety was observed among individuals undergoing 24-hour Holter recording. Anxiety was significantly associated with self-reported cardiac symptoms, though not with the presence of arrhythmias. The findings could have practical implications for clinical practice. The study suggests that patients reporting anxious palpitations should receive a more thorough cardiological assessment, with 24-hour Holter monitoring serving as an effective tool for this evaluation.

## In Brief

Depression, anxiety, distress and Type D personality traits have been implicated in the pathogenesis of cardiovascular diseases. Mental health status is associated with arrhythmic events. Esler and Lampert reported that anxiety and distress contribute to the occurrence of atrial and ventricular arrhythmias.

## Highlights

■ Self-reported cardiac symptoms not associated with arrhythmias.

■ High prevalence of anxiety and depression symptoms.

■ Anxiety was correlated with self-reported cardiac symptoms.

This article is part of author Renata Lima Giolo’s master’s thesis defended in 2020 by the *Faculdade Israelita de Ciências da Saúde Albert Einstein*, São Paulo, Brazil.

## Graphical abstract

**Figure:**
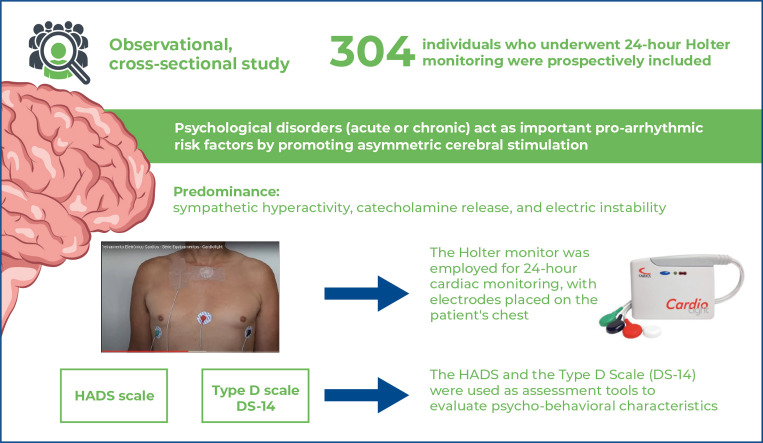

## INTRODUCTION

Cardiovascular disease (CVD) is a major public health issue worldwide, causing 20.5 million deaths per year.^1^ Prevention strategies and the identification of risk factors are essential for mitigating the disease’s burden. In addition to traditional risk factors, such as high cholesterol, hypertension, and diabetes, psychological factors have also been implicated in CVD development.^2^

Depression, anxiety, distress (negative stress), and Type D (distressed) personality profiles have been identified as contributors to the pathogenesis of CVD, influencing both treatment outcomes and prognosis.^3-5^ Mental health status is associated not only with atherosclerotic burden but also with arrhythmic events. For instance, anxiety and distress are linked to the occurrence of atrial and ventricular arrhythmias.^6,7^ Psychological disorders, both acute and chronic, are crucial proarrhythmic risk factors. They promote asymmetric cerebral stimulation primarily affecting the right hemisphere, which increases sympathetic hyperactivity, catecholamine release, and electrical instability within the cardiac conduction system.^8,9^

In this context, 24-hour Holter monitoring serves as a valuable diagnostic tool for detecting arrhythmic events while simultaneously assessing self-reported symptoms. Patients frequently associate anxiety and stress with palpitations and tachycardia; however, the interaction between these psychological factors and arrhythmic events has not been fully explored. Few studies have conducted multidimensional psycho-behavioral analyses in individuals undergoing 24-hour Holter monitoring.

This type of assessment could help clarify the relationship between psychological factors and self-reported arrhythmic events, regardless of their confirmation by electrocardiography (ECG). We hypothesized that psychological conditions or traits, such as anxiety, depression, distress, and Type D personality, might influence the perception of cardiac symptoms in individuals undergoing 24-hour Holter monitoring.

## OBJECTIVE

To examine the presence of psychological conditions or traits and their potential association with the perception of cardiac symptoms in individuals undergoing 24-hour Holter monitoring.

## METHODS

### Study design

This cross-sectional observational study involved 304 consecutive individuals who underwent 24-hour Holter monitoring from May 4, 2017, to August 18, 2018. This study was approved by the Institutional Review Board of *Hospital Israelita Albert Einstein* (CAAE: 63181416.1.0000.0071; # 1.980.308, SGPP: 2882-16).

The inclusion criteria were as follows: individuals of both sexes, aged over 18 years, scheduled for 24-hour Holter monitoring after an outpatient consultation as per the attending physician’s recommendation. The exclusion criteria were as follows: inpatient, individual with visual impairment, illiteracy, difficulty in understanding the assessment questionnaires, or non-Portuguese speaker. The study adhered to Strengthening the Reporting of Observational Studies in Epidemiology guidelines for transparent reporting.^10^

### Measures

Individuals were invited to participate and enrolled after Holter monitoring installation and providing informed consent. Clinical and demographic data were collected upon consent. Psycho-behavioral assessment was conducted using the following questionnaires:

1. Hospital Anxiety and Depression ScaleHADS (Brazilian version) - A 14-item scale with seven items each for anxiety and depression symptoms, rated as mild, moderate, or severe. Scores ranging from 8 to 21 indicate the presence of symptoms. When used for stress assessment, all 14 items are summed, with scores >15 indicating the presence of stress symptoms.^11,12^
2. Type D Scale (DS-14; Brazilian version) - A Type D 14-item personality scale divided into two subscales assessing negative affectivity and social inhibition. Positive cut-off scores of ≥10 on both subscales indicate Type D personality.^13,14^

Ambulatory electrocardiographic monitoring was performed using the CardioLight^®^; CARDIOS, Brazil, device with a 24-hour, 3-channel Holter system. Participants were provided with a diary to record their symptoms. The recordings were classified according to baseline rhythm (sinus rhythm, ectopic atrial rhythm, pacemaker rhythm, atrial tachycardia, atrial fibrillation, or atrial flutter) and by the presence and density of arrhythmias (premature contractions (PCs) and tachycardia), following the criteria adapted from Myerburg et al.: nil, rare (<1 arrhythmias/hour), infrequent (10-29 arrhythmias/hour), or frequent (≥30 arrhythmias/hour).^15^

Furthermore, the recorded symptoms were classified as cardiac (palpitations, skipped heartbeats, chest pain, presyncope, and syncope) or non-cardiac (tingling, nausea, headache, shaking, blurred vision, and nonspecific discomfort). Participants also recorded the absence of symptoms in the diary. For the analysis, the participants were divided into two groups: those with cardiac symptoms and those with non-cardiac or no symptoms (asymptomatic).

### Statistical analyses

Data are presented as frequencies (%) and means (standard deviation). Baseline and electrocardiographic variables were compared between the participants with and without self-reported cardiac symptoms using χ^2^ test, unpaired Student’s *t*-test, and Fisher’s Exact test.

A logistic regression model adjusted for age, sex, physical activity (yes/no), employment status (active/inactive), marital status, presence of hypertension, history of arrhythmia, undergone heart transplantation and/or being on transplant list, presence of hypothyroidism or hyperthyroidism, psychotropic medication use, and baseline rhythm during 24-hour Holter monitoring (atrial fibrillation, atrial flutter, or the presence of frequent or infrequent PCs) was used to assess the association between psychological characteristics and self-reported cardiac symptoms. Statistical significance was set at p<0.05. All statistical analyses were performed using SPSS version 20, IBM.

## RESULTS

Table 1 presents the clinical and demographic characteristics in relation to the presence of cardiac symptoms. The distribution of participants based on sex (male/female) was balanced and the mean age was lower than that observed in other studies on this topic.

**Table 1:** Demographic and clinical characteristics of the participants in relation to self-reported cardiac symptoms during 24-hour Holter monitoring

Variable	Self-reported cardiac symptoms		Total	p value
	No	Yes		
	n=254	n=50		
Sex				
Female	128 (50.4)	26 (52)	154 (50.7)	0.835
Age, years	50.43±16.39	46.38±12.72	49.76±15.90	0.054
Education, n (%)				0.594
Elementary	3 (1.2)	1 (2)	4 (1.3)	
High school	59 (23.3)	9 (18)	68 (22.4)	
University or College	149 (58.7)	31 (62)	180 (59.2)	
Post Graduate Education	43 (16.8)	9 (18)	52 (17.1)	
Professional status, n (%)				0.063
Employed	173 (68.1)	41 (82)	214 (70.4)	
Away from work	4 (1.6)	1 (2)	5 (1.6)	
Unemployed	1 (0.4)	0 (0)	1 (0.3)	
Retired	47 (18.5)	2 (4)	49 (16.1)	
Not active	29 (11.4)	6 (12)	35 (11.6)	
Existing clinical disease or health status, n (%)				
Heart disease	64 (25.2)	11 (22)	75 (24.7)	0.513
Hypertension	96 (37.8)	11 (22)	107 (35.2)	0.033
Diabetes	34 (13.4)	4 (8)	38 (12.5)	0.293
Arrhythmia	71 (28)	19 (38)	90 (29.6)	0.155
Cardiac surgery/PCI	19 (7.5)	4 (8)	23 (7.6)	>0.999*
Ablation/ECV	10 (3.9)	3 (6)	13 (4.3)	0.455*
Transplantation/Waiting list	21 (8.3)	7 (14)	28 (9.2)	0.191*
Hypothyroidism/ hyperthyroidism	42 (16.5)	13 (26)	55 (18.1)	0.112
Tobacco use	17 (6.7)	3 (6)	20 (6.6)	>0.999*
Alcohol use	142 (55.9)	29 (58)	171 (56.3)	0.785
Physical activity	136 (53.5)	22 (44)	158 (52)	0.217

Values in each column represent the number of patients, accompanied by the percentage (%) relative to the total number of patients. Age data are presented as mean ± SD. P-values were calculated using the χ^2^ test or * Fisher’s Exact test.

PCI: percutaneous coronary intervention; ECV: electrical cardioversion.

One-quarter of the participants (n=76) reported using psychotropic medication, with antidepressants being the most common (12.5%). Additionally, 50 participants (16.4%) reported cardiac-related symptoms.

The primary indication for Holter monitoring was treatment assessment (37%) in participants with a history of arrhythmias, cardiomyopathies, or cardiac surgery. Electrocardiography revealed normal sinus rhythm in 89.8% of the participants. Paroxysmal atrial fibrillation was observed in 1.6% of the participants.

The most common arrhythmias were occasional PCs with a low incidence (<10 PCs/hour). During Holter monitoring, premature ventricular contractions were observed in 53.5% of the participants who did not self-report cardiac symptoms and in 60% of those who did, while premature atrial complexes were observed in 71.7% of the participants without cardiac symptoms and 74% of those with symptoms. No significant difference in arrhythmic events was found between participants who did or did not self-report cardiac symptoms during Holter monitoring (Table 2).

**Table 2:** Distribution of arrhythmias according to density categories and self-reported cardiac symptoms during 24-hour Holter monitoring

Variable	Self-reported cardiac symptoms		Total	p value
	No n=254	Yes n=50		
PVCs, n (%)				0.653
Nil	67 (26.4)	10 (20)	77 (2.3)	
Rare (<1 PVCs/hour)	136 (53.5)	30 (60)	166 (54.6)	
Infrequent (10-29 PVCs/hour)	14 (5.5)	4 (8)	18 (6)	
Frequent (≥ 30 PVCs/hour)	37 (14.6)	6 (12)	43 (14.1)	
PACs, n (%)				0.142
Nil	39 (15.4)	6 (12)	45 (14.8)	
Rare (<1 PACs/hour)	182 (71.7)	37 (74)	219 (72)	
Infrequent (10-29 PACs/hour)	11 (4.3)	0 (0.0)	11 (3.7)	
Frequent (≥ 30 PACs/hour)	22 (8.6)	7 (14)	29 (9.5)	
NSVT, n (%)				>0.999
Nil	233 (91.7)	46 (92)	279 (91.8)	
Rare (<1 NSVT/hour)	21 (8.3)	4 (8)	25 (8.2)	
NSAT, n (%)				0.834
Nil	188 (74)	37 (74)	225 (74)	
Rare (<1 NSAT/hour)	65 (25.6)	13 (26)	78 (25.7)	
Infrequent (10-29 NSATs/hour)	1 (0.4)	0 (0.0)	1 (0.3)	

Values in each column represent the number of patients, accompanied by the percentage (%) relative to the total number of patients. P-values were calculated using Fisher’s Exact test.

PVCs: premature ventricular complexes; PACs: premature atrial complexes; NSVT: non-sustained ventricular tachycardia; NSAT: non-sustained atrial tachycardia.

Among the participants, only 13.1% described symptoms that correlated with significant electrocardiographic changes.

Psychological factors related to self-reporting of cardiac symptoms were assessed using the HADS and DS-14 scales (Table 3). No significant differences were found between the self-reported and non-self-reported groups in the prevalence of any of the four psychological traits. Although not statistically significant, a trend toward a higher prevalence of anxiety symptoms was observed in the self-reported group (p=0.060).

**Table 3:** Psychological characteristics assessed by HADS and DS-14 questionnaires in individuals with and without self-reported cardiac symptoms during 24-hour Holter monitoring

Variable	Self-reported cardiac symptoms		Total	p value
	No n=254	Yes n=50		
HADS anxiety, n (%)				0.060
No anxiety	150 (59)	24 (48)	174 (57.2)	
Mild anxiety	57 (22.4)	17 (34)	74 (24.4)	
Moderate anxiety	44 (17.4)	6 (12)	50 (16.4)	
Severe anxiety	3 (1.2)	3 (6)	6 (2)	
Total individuals with anxiety	130 (42.7)			
HADS depression, n (%)				0.570
No depression	215 (84.6)	43 (86)	258 (85)	
Mild depression	26 (10.3)	3 (6)	29 (9.5)	
Moderate depression	12 (4.7)	4 (8)	16 (5.2)	
Severe depression	1 (0.4)	0	1 (0.3)	
Total individuals with depression	46 (15.1)			
HADS distress, n (%)				0.684
No	186 (73.2)	38 (76)	224 (73.7)	
Yes	68 (26.8)	12 (24)	80 (26.3)	
Type D personality, n (%)				0.565
No	207 (81.5)	39 (78)	246 (81)	
Yes	47 (18.5)	11 (22)	58 (19)	

Values in each column represent the number of patients, accompanied by the percentage (%) relative to the total number of patients. P-values were calculated using the χ^2^ test.

HADS: Hospital Anxiety and Depression Scale.

To further explore the association between anxiety and self-reported cardiac symptoms, a logistic regression model was applied during 24-hour Holter monitoring. As shown in table 4, both mild and severe anxiety were significantly associated with an increased likelihood of reporting cardiac symptoms (odds ratio (OR) = 2.305, 95%CI= 1.098-4.841, p=0.027 and OR= 9.245, 95%CI= 1.582-54.013, p=0.014, respectively), even after adjusting for potential confounders, such as the presence of arrhythmia. Furthermore, participants with a heart transplant, those on the transplant waiting list, or those with unstable arrhythmias were more likely to self-report cardiac symptoms during the 24-hour Holter monitoring (Table 4). In contrast, being inactive in their occupation or being hypertensive was associated with a lower likelihood of self-reporting cardiac symptoms.

**Table 4:** Adjusted logistic regression analysis showing the association between anxiety symptoms and the self-reported cardiac symptoms during 24-hour Holter monitoring (final model)

Variable	OR	95%CI	p value
HADS anxiety (ref.: No anxiety)			
Mild anxiety	2.305	1.098-4.841	0.027
Moderate anxiety	0.787	0.29-2.135	0.639
Severe anxiety	9.245	1.582-54.013	0.014
Professional status (inactive)	0.299	0.122-0.734	0.008
Hypertension	0.398	0.182-0.871	0.021
Transplantation/Waiting list	3.139	1.077-9.149	0.036
Hypothyroidism/Hyperthyroidism	2.027	0.941-4.366	0.071
PAF/infrequent or frequent PC	2.162	1.039-4.496	0.039

Model adjusted for sex, age, physical activity, professional status, marital status, hypertension, prior arrhythmia, transplantation or waiting list, hypothyroidism/hyperthyroidism, use of psychotropics, PAF, infrequent and frequent PCs, and HADS anxiety score.

HADS: Hospital Anxiety and Depression Scale; OR: odds ratio; 95%CI: 95% confidence interval; PAF: paroxysmal atrial fibrillation; PC: premature contractions.

Logistic regression models examining the relationship between symptoms of depression, distress, or Type D personality, and the self-reporting of cardiac symptoms did not yield statistically significant results.

## DISCUSSION

### Main findings

Most self-reported cardiac symptoms during Holter monitoring were not corroborated by corresponding ECG patterns. However, regression analysis indicated that individuals with anxiety symptoms were more likely to self-report non-ECG-verifiable cardiac symptoms. Participants with pre-existing heart disease or those who had undergone heart surgery or were on a transplant waiting list were more likely to report cardiac symptoms. Overall, anxiety symptoms were observed in 42.7% of enrolled participants, while depression symptoms were observed in 15.1%. Mild/moderate anxiety symptoms were observed in 46% of the participants, while mild/moderate depression in 14%. Few studies have explored the relationship between psychological profiles and cardiac symptoms using 24-hour Holter monitoring.^16,17^

Mild and severe anxiety symptoms were associated with an increased likelihood of detecting cardiac symptoms on the 24-hour Holter monitor. However, the current data on the relationship between anxiety and arrhythmias remain inconclusive.^4,7,18,19^ Some meta-analyses have identified anxiety symptoms, generalized anxiety disorder, panic disorder, and post-traumatic stress syndrome as risk factors for cardiovascular events, yet the direct relationship between anxiety and arrhythmias remains unclear.^20,21^

The prevalence of anxiety is notably high in patients with implantable cardioverter-defibrillator (ICD), though no study has definitively established the relationship between prevalence of anxiety and the occurrence of ventricular arrhythmias.^18^ Our findings suggest that anxiety symptoms may explain the reporting of cardiac symptoms, regardless of the presence of arrhythmia.

The observed prevalence of depressive symptoms was higher than that previously reported for the general population.^22,23^ The comparison of these data with our results is limited because our study assessed depressive symptoms rather than clinical depression. Notably, the prevalence of depression is high in patients with CVD, particularly those who are hospitalized, where it adversely affects quality of life, adherence to treatment, and increases the risk of cardiovascular events. Patients with depressive symptoms tend to experience fewer symptoms, such as palpitations and dyspnea, which can directly impact their care. This reduced symptom recognition may prevent them from identifying signs of clinical deterioration and seeking timely medical help.^24-26^

The proarrhythmic effects of antidepressants, particularly tricyclics and selective serotonin reuptake inhibitors, remain uncertain. Evidence suggests that their use may increase the risk of atrial fibrillation.^27^

Palpitations are a common complaint in clinical practice, and can be triggered by distress. Studies have linked distress to arrhythmic events, both acute (sustained and non-sustained ventricular arrhythmias documented in patients with ICD) and chronic (occurring after ischemic events and episodes of paroxysmal atrial fibrillation).^17,28-32^ However, despite distress being observed in a quarter of the study sample, no association was found between distress and cardiac symptoms, which could be attributed to the heterogeneous nature of the samples.

Similarly, Type D personality did not correlate with cardiac symptoms in our study. Previous research using Holter monitoring reported a higher likelihood of arrhythmias (ventricular bigeminism and non-sustained ventricular arrhythmias) in Type D individuals; however, no association was observed with the presence of symptoms.^33^

Our findings highlight the importance of detailed cardiologic evaluation for individuals reporting palpitations associated with anxiety, with a 24-hour Holter being an effective tool for this evaluation. Additionally, psycho-behavioral assessments of these individuals are crucial for clarifying symptoms and guiding behavior, which may extend beyond arrhythmia treatment to address psychological triggers or contributors. In these cases, controlling factors that can act as triggers or retro-feeders of arrhythmias is necessary as a complementary measure to treatment.

The limitations of this study include its design, which did not allow causal inferences. In addition, the single-center nature of the study and the heterogeneity of the sample warrant caution when generalizing the results. Furthermore, the self-reported nature of the assessment instruments introduces potential bias as participants may provide socially desirable responses or align their answers with perceived expectations of the researchers.

## CONCLUSION

In this study, most self-reported cardiac symptoms during 24-hour Holter monitoring could not be corroborated by corresponding electrocardiogram patterns. Regression analysis demonstrated that individuals with anxiety symptoms were more likely to self-report cardiac symptoms that were not verifiable by the electrocardiogram.
